# A Novel Artificial Intelligence-Powered Emotional Intelligence and Mindfulness App (Ajivar) for the College Student Population During the COVID-19 Pandemic: Quantitative Questionnaire Study

**DOI:** 10.2196/25372

**Published:** 2021-01-05

**Authors:** Ronda Sturgill, Mary Martinasek, Trine Schmidt, Raj Goyal

**Affiliations:** 1 Department of Health Sciences and Human Performance The University of Tampa Tampa, FL United States; 2 Ajivar Tarpon Springs, FL United States

**Keywords:** mindfulness, COVID-19, college students, emotional intelligence

## Abstract

**Background:**

Emotional intelligence (EI) and mindfulness can impact the level of anxiety and depression that an individual experiences. These symptoms have been exacerbated among college students during the COVID-19 pandemic. Ajivar is an app that utilizes artificial intelligence (AI) and machine learning to deliver personalized mindfulness and EI training.

**Objective:**

The main objective of this research study was to determine the effectiveness of delivering an EI curriculum and mindfulness techniques using an AI conversation platform, Ajivar, to improve symptoms of anxiety and depression during this pandemic.

**Methods:**

A total of 99 subjects, aged 18 to 29 years, were recruited from a second-semester group of freshmen students. All participants completed the online TestWell Wellness Inventory at the start and end of the 14-week semester. The comparison group members (49/99, 49%) were given routine mental wellness instruction. The intervention group members (50/99, 51%) were required to complete Ajivar activities in addition to routine mental wellness instruction during the semester, which coincided with the onset of the COVID-19 pandemic. This group also completed assessments to evaluate for anxiety, using the 7-item Generalized Anxiety Disorder (GAD-7) scale, and depression, using the 9-item Patient Health Questionnaire (PHQ-9).

**Results:**

Study participants reported a mean age of 19.9 (SD 1.94) years; 27% (27/99) of the group were male and 60% (59/99) identified as Caucasian. No significant demographic differences existed between the comparison and intervention groups. Subjects in the intervention group interacted with Ajivar for a mean time of 1424 (SD 1168) minutes. There was a significant decrease in anxiety, as measured by the GAD-7: the mean score was 11.47 (SD 1.85) at the start of the study compared to 6.27 (SD 1.44) at the end (*P*<.001). There was a significant reduction in the symptoms of depression measured by the PHQ-9: the mean score was 10.69 (SD 2.04) at the start of the study compared to 6.69 (SD 2.41) at the end (*P*=.001). Both the intervention and comparison groups independently had significant improvements in the TestWell Wellness Inventory from pretest to posttest. The subgroups in the social awareness and spirituality inventories showed significant improvement in the intervention group. In a subgroup of participants (11/49, 22%) where the GAD-7 was available during the onset of the COVID-19 pandemic, there was an increase in anxiety from the start of the study (mean score 11.63, SD 2.16) to mid-March (ie, onset of the pandemic) (mean score 13.03, SD 1.48; *P*=.23), followed by a significant decrease at the end of the study period (mean score 5.9, SD 1.44; *P*=.001).

**Conclusions:**

It is possible to deliver EI and mindfulness training in a scalable way using the Ajivar app during the COVID-19 pandemic, resulting in improvements in anxiety, depression, and EI in the college student population.

## Introduction

### Background

Students who do not perform well at college often have low emotional intelligence (EI), even students with a high IQ. EI involves a set of learned noncognitive skills, which helps individuals foster better relationships, improve time and stress management, maintain better impulse control, and improve problem-solving abilities [1-3]. Mindfulness has been defined as the awareness that arises by “paying attention to the present moment on purpose and non-judgmentally” [4]. EI and mindfulness can impact the level of anxiety and depression an individual experiences. This is particularly important during stressful times, such as during the COVID-19 pandemic. Ajivar is an app that utilizes artificial intelligence (AI) and machine learning (ML) to deliver personalized mindfulness and EI training to college students. Through ongoing engagement of the user, the app can decrease stress and anxiety while improving performance and emotional well-being.

Anxiety and depression are ever prominent among college students. More than 50% of the 20 million college students in the United States reported symptoms of anxiety and depression that prevented them from performing well at college [5]. The symptoms associated with anxiety and depression often persist over time. More than 75% to 85% of college students have reported feeling stressed and overwhelmed [6-8]. There are several reasons college students experience disproportionately higher levels of stress than the general population, including first-time independence leading to having to manage their academic responsibilities, financial responsibility, and planning for their future [9,10]. Despite the prevalence and persistence of anxiety and depression, up to 75% of college students do not access the needed resources and needed assistance, primarily because of the underlying stigma associated with a diagnosis and mental health services [11]. Additionally, colleges and universities have limited resources to aid the large number of students who suffer from mental health conditions. These conditions are further exacerbated by extenuating circumstances, such as the COVID-19 pandemic.

### COVID-19

On March 11, 2020, the World Health Organization declared COVID-19, an acute respiratory syndrome caused by SARS-CoV-2, a global pandemic [12]. Not only has this novel pandemic resulted in severe health consequences, but it has also impacted the mental well-being of college students who have struggled with immediate displacement from college, learning online models of course delivery, and having to socially distance from their friends and social support. In a self-reported survey by the Healthy Minds Network during the COVID-19 crisis, college students reported increased mental health concerns (ie, anxiety, depression, suicidal ideation, etc), which resulted in poor academic performance [13]. Increased frequency of anxiety and depression have been reported among undergraduate students during the pandemic [14,15].

Initial findings from the pandemic suggest that the mental health consequences will be far reaching. Time will tell the true impact of the social and behavioral changes experienced by college students. Providing novel, scalable, and disruptive tools to help this vulnerable section of the population is going to be important if we are to prevent long-term mental health sequelae from the pandemic.

### Emotional Intelligence and Mindfulness

EI is “a set of skills hypothesized to contribute to the accurate appraisal and expression of emotion in oneself and in others, the effective regulation of emotion in self and others, and the use of feelings to motivate, plan, and achieve in one’s life” [16]. The ability to accurately identify, manage, and express emotions is the foundation of good self-esteem, healthy relationships, and good work and academic performance [17]. EI is not commonly taught in colleges and universities, which can inhibit students’ full potential of learning due to lack of coping skills in managing stress, anxiety, and interpersonal relationships. The inability to emotionally self-regulate often leads to psychopathology due to the avoidance of, or preoccupation with, negative emotion [18] and, as a result, may have negative consequences on the individual’s health, relationships, and work and school performance [19]. EI acts as a protective factor for mental health and is positively associated with adaptive coping styles, peer relationships, and socioemotional competence [20]. EI also mediates the relationship between mindfulness and depression and anxiety in adolescents [21].

Mindfulness is a state of mind, a moment-to-moment awareness of one’s experience without judgment [22]. Being fully focused on the present moment allows for the absence of rumination and negative thoughts that lead to stress and anxiety. Mindfulness positively correlates with the capacity to be more emotionally aware, including the ability to identify and change emotional states [23]. Several mindfulness techniques, such as meditation, have proven efficacy and have shown a reduction in depression, anxiety, and rumination [24]. Mindfulness has also been linked to emotional stability, such as calmness, clarity, and concentration, as well as to emotion regulation [25]. Bridging mindfulness and EI is positive psychology, the science of happiness and flourishing [26]. It is a strength-based therapeutic approach to mental health that highlights well-being, resiliency, and compassion, which are also key factors in Ajivar.

Previous studies utilizing different mobile apps to deliver mindfulness and EI have proven effective in a myriad of populations. Improvements in psychosocial outcomes, an increase in the effectiveness of mindfulness treatment, and an increase in positive psychological interventions were all found in adult populations following the use of mindfulness apps [27-29].

### Study Objectives

The main objective of this research study was to determine the effectiveness of delivering an EI curriculum and mindfulness techniques using an AI conversation platform, Ajivar. Several validated and self-reported measurements of mental wellness and EI were tracked, including the TestWell Wellness Inventory, the 7-item Generalized Anxiety Disorder (GAD-7) scale, the 9-item Patient Health Questionnaire (PHQ-9), and the emotional quotient (EQ).

The study was launched at the end of January 2020; then in March, the COVID-19 pandemic began to impact students, staff, and faculty. Another objective that presented itself because of the pandemic was to evaluate how EI and mindfulness techniques taught during Ajivar interactions impacted student mental wellness and EI. The timing of the research study offered us an opportunity to evaluate the app during a pandemic in this population.

## Methods

### Study Design

Subjects included students attending a midsized liberal arts institution in Florida, United States. The students in the sample were attending the university as second-semester freshmen. This study consisted of comparison (49/99, 49%) and intervention (50/99, 51%) groups. All participants were college-age freshmen, who were 18 to 29 years of age, and were given routine mental wellness instructions as part of the classes.

Students in the intervention group were required to complete Ajivar activities as part of the course requirement. The requirement included a minimum of two Ajivar interactions per week. The comparison group members did not utilize Ajivar during the semester. All subjects completed the TestWell Wellness Inventory at the beginning and end of the 14-week semester. Completion of the inventory was a course requirement in the intervention and comparison groups. During interactions with Ajivar, the students completed assessment questionnaires as outlined below.

### Assessment Questionnaires

#### The TestWell Wellness Inventory

The TestWell Wellness Inventory [30,31] for college students consists of 100 questions on a 5-point scale. The questions included assessment of physical fitness, self-care and safety, social awareness, emotional management, occupational wellness, nutrition, environmental wellness, emotional wellness, emotional awareness, intellectual wellness, spirituality, and values.

#### The 9-Item Patient Health Questionnaire

The PHQ-9 is a valid, reliable, 9-item self-administered questionnaire that evaluates the past 2 weeks’ depressive symptoms using a scale from 0 to 3 per item [32]. The total score is divided into four outcome ranges of depression severity: 0-4 (no symptoms), 5-9 (mild depression), 10-14 (moderate depression), 15-20 (moderate-severe depression), and 21-27 (severe depression) [32].

#### The 7-Item Generalized Anxiety Disorder Scale

The GAD-7 scale is a valid, reliable, 7-item self-administered questionnaire that is used as a screening tool and severity measure for generalized anxiety disorder [33]. The scores range from 0 to 21 and are categorized according to symptom severity: 0-4 (minimal anxiety), 5-9 (mild anxiety), 10-14 (moderate anxiety), and 15-21 (severe anxiety) [33].

#### Emotional Quotient

There are several measures of EI [17]. As part of the Ajivar EI curriculum that is delivered via the app, there is a proprietary self-reported EQ assessment. This EQ assessment evaluates the user across several categories, which include self-awareness, self-regulation, self-efficacy, empathy, and social skills. The score is normalized to 100, and scores are characterized as follows: ≤39 (low EI), 40-49 (low-average EI), 50-69 (average EI), and ≥70 (high EI).

### Ajivar: An EI- and AI-Powered Life Coach

Using AI and ML, Ajivar delivers personalized EI training and mindfulness techniques through brief conversations, videos, and activities founded in self-help practices from positive psychology and mindfulness. The Ajivar platform interacts with the user in a text-based conversational format, similar to interactions users can have with Alexa, Google Assistant, Cortana, etc. During these interactions, the information gathered by Ajivar helps it learn about the individual and the community the individual belongs to. The Ajivar app provides fun and uplifting ways to engage the user, such as positive affirmations (ie, “Posimations”), journaling, and out-of-zone (ie, “Ooz”) challenges that help people get outside of their comfort zone to increase self-esteem and acceptance.

Ajivar delivers a personalized EI curriculum and mindfulness techniques to students that are approved by the Ajivar clinical consultation team. The app acts as a life coach that helps the user improve self-esteem and social and emotional awareness. Ajivar responds in a text-based conversation to emotions and underlying beliefs that the user expresses during the interactions with the app. Using emotionally intelligent conversations, the app supports and guides the user with empathetic responses, individualized content, and feedback and encourages the individual to apply their knowledge outside of the app. Apart from the psychoeducational content of positive psychology and mindfulness, the app also utilizes the following components to improve EI and resiliency:

1. Personalization (ie, automated tailoring). Ajivar utilizes ML that is based on all users’ interactions, in addition to the specific user’s interactions, to deliver personally relevant information and techniques based on what would benefit the user in the specific moment.
2. Resiliency and EI training. The app evaluates the user’s current level of EI (ie, EQ score) based on built-in assessment tools and the user’s responses during interactions. Ajivar utilizes an EI curriculum developed by clinicians to build coping skills and resiliency that are used to form healthy habits in the real world.
3. Reflection. The journaling component of the app is used for gratitude practice and reflecting on insights and feelings. The sentiment analysis function provides the user with feedback on their past and current emotional state. This insight is imperative to reinforce learning and growth.
4. Positive affirmations. These are personalized through the app interactions and are stored in a list for the user to review on a regular basis to combat negative self-talk.
5. Engagement. Ajivar uses real-time engagement, such as notifications, challenges, app mentors (ie, avatars that are collected), access to videos, and other gamification tools to keep the user engaged. The AI also gives feedback on mood and emotions, which provides the users with real-time feedback on the dashboard.

The app can be downloaded from the Apple App Store and Google Play. All interaction data are encrypted and stored on a separate server from any identifiable personal information. Only anonymized data were used for research analysis. Ajivar is not a medical device and is designed to be a support tool used for mental well-being. Anonymized data regarding the user interactions were provided to the researchers. This included the duration of user interactions with the different components of the Ajivar app and the number of interactions that took place during the study time interval.

### Quantitative Measurements

The data were analyzed using the statistical software SPSS, version 26 (IBM Corp) [34].

### User Engagement and Attrition

There were a total of 93 students who completed the TestWell pretest and posttest: 49 participants in the comparison group (53%) and 44 participants in the intervention group (47%).

In the intervention group, 49 out of 50 participants (98%) downloaded and interacted with the Ajivar app. If a participant carried out a greater level of activity than the course requirements, they were categorized as *high engagers* (30/49, 61%). Participants that carried out the required level of activity or less than what was required by the course were identified as *low engagers* (19/49, 39%). The mean total time on the app for the high engagers was 2069 (SD 1076) minutes versus 480 (SD 321) minutes among the low engagers. Mean total engagement for the group was 1424 (SD 1168) minutes.

### Analysis of Assessment Questionnaires

An analysis of covariance (ANCOVA) was used to evaluate the TestWell pre- and posttest scores for the intervention group, the comparison group, and the individual subgroups. Pre- and posttest scores for the GAD-7, the PHQ-9, and the EQ were evaluated for differences over time for the high engagers. A GAD-7 time series of pretest, midpoint (ie, March 2020), and posttest scores was also evaluated.

### Informed Consent and Institutional Review Board Approval

The study was reviewed and approved by the Institutional Review Board at the University of Tampa. Participation was voluntary, and the participants indicated their consent to the study protocol via the informed consent form. All study data were collected by the academic institution with the exception of the Ajivar app usage data. All usage data were anonymized before being given to the research team. Usage data were not linked to specific research participants.

## Results

### Demographic Data

This study consisted of comparison (49/99, 49%) and intervention (50/99, 51%) groups. The mean age of the study participants who reported was 19.9 (SD 1.94) years; 27% (27/99) were male and 69% (68/99) were female. Over half the students reported their ethnicity as Caucasian (59/99, 60%) and the rest reported their ethnicity as Hispanic (13/99, 13%), African American (7/99, 7%), Asian Pacific Islander (7/99, 7%), and Other (13/99, 13%). No significant demographic differences existed between the comparison and intervention groups.

### Quantitative Analysis

#### Analysis of Assessment Questionnaires

To answer the first research question regarding the effectiveness of the TestWell Wellness Inventory, ANCOVA was performed to assess whether statistically significant differences occurred between the intervention and comparison groups based on their pre- and posttest scores on the Wellness Inventory. The results were not statistically significant (*P*=.41). However, both the intervention group (*P*=.04) and the comparison group (*P*=.002) independently showed statistically significant improvements from pretest to posttest on the Wellness Inventory.

The Wellness Inventory was assessed further using ANCOVA for statistically significant differences between the intervention group and the comparison group pretest and posttest on subgroups. In the intervention group, the inventory values for the social awareness subgroup significantly improved from pretest to posttest, as did the values for the spirituality subgroup. The results are shown in Table 1.

**Table 1:** Analysis of covariance of TestWell Wellness Inventory scores for each subgroup.

Subgroup	TestWell Wellness Inventory scores, mean (SD)		*P* value
	Pretest	Posttest	
Social awareness	82.32 (12.56)	88.91 (10.89)	.04
Spirituality	83.32 (13.86)	88.23 (12.74)	.04

#### Mental Wellness Indicators

In the intervention group’s high-engagers subset (30/49, 61%), we assessed mean differences between the pre- and posttest scores for GAD-7, PHQ-9, and EQ. The results were found to be statistically significant as noted in Table 2. In particular, mean GAD-7 scores decreased from 11.47 (SD 1.85) to 6.27 (SD 1.44) (*P*<.001), indicating a statistically significant decline in anxiety from the beginning to the end of the study. Mean PHQ-9 scores decreased significantly from 10.69 (SD 2.04) to 6.69 (SD 2.41) (*P*<.001), indicating improved mental health in this aggregate data. EQ scores increased from pre- to poststudy, with initial mean aggregate scores of 62.87 (SD 10.12) that increased to 71.17 (SD 8.46) (*P*<.001).

**Table 2:** Mean differences between the 7-item Generalized Anxiety Disorder (GAD-7) scale, the 9-item Patient Health Questionnaire (PHQ-9), and the emotional quotient (EQ).

Measure	Mean (SD)	Mean difference	*P* value
GAD-7 pretest	11.47 (1.85)	5.2	<.001
GAD-7 posttest	6.27 (1.44)	N/A^a^	N/A
PHQ-9 pretest	10.69 (2.04)	4.0	<.001
PHQ-9 posttest	6.69 (2.41)	N/A	N/A
EQ pretest	62.87 (10.12)	–8.3	<.001
EQ posttest	71.17 (8.46)	N/A	N/A

^a^N/A: not applicable.

#### COVID-19 Impact

**Figure 1:**
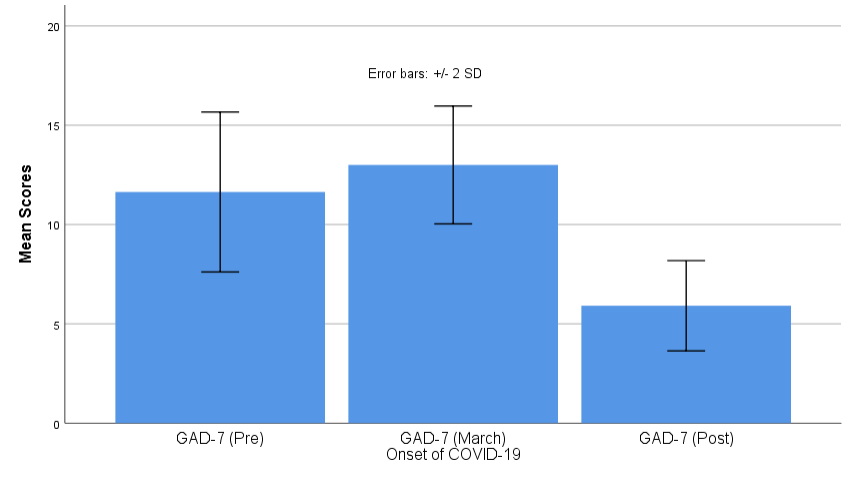
Repeated-measures analysis of variance for within-subjects differences in 7-item Generalized Anxiety Disorder (GAD-7) scale scores.

We collected midpoint data in the spring semester on GAD-7 scores. We conducted repeated-measures analyses of variance to assess within-subjects differences in this intervention subgroup (11/49, 22%). The GAD-7 results indicated a mean pretest (time 1) score of 11.64 (SD 2.16), a mid-March (time 2) mean score of 13.03 (SD 1.48), and an end-of-spring (time 3) mean score of 5.9 (SD 1.44) (see Figure 1). The Greenhouse-Geisser correction indicated statistically significant (*P*=.001) within-subjects differences on the continuous variable. Bonferroni pairwise comparison corrects for multiple comparisons; the results indicated statistical significance between times 1 and 3 and times 2 and 3, but not between times 1 and 2.

## Discussion

### Principal Findings

The findings from our study suggest that the use of Ajivar had many positive benefits for the college student participants. These results were especially impactful given that the students experienced the pandemic during the course of the study. We found that students in the intervention group had less depression over time as measured by the PHQ-9, less anxiety as measured by the GAD-7, and improvement in EI as measured by the EQ. Student anxiety increased around mid-March and then decreased significantly by the end of the semester, despite the upheaval caused by the pandemic. Mid-March was the time during the semester when learning was shifted for all students at the university to a remote format. This transition occurred over the course of a few days. Further testing is suggested as the pandemic continues. The subgroups of social awareness and spirituality were significantly improved with the use of the app. There were also areas of no significance, including the pretest and posttest scores of the TestWell Wellness Inventory. We also noted a high engagement rate, with over 60% of users interacting with the Ajivar app for a greater amount of time than was required.

### Comparison With Prior Work

Previous studies utilizing different mobile apps to deliver mindfulness and EI techniques have proven effective in a myriad of populations. Improvements in psychosocial outcomes were noted with mindfulness training and positive psychological interventions in adult populations following the use of mindfulness apps [27-29]. Champion et al showed a significant improvement of self-reported stress, resilience, and satisfaction with life among adults after 10 days of using a mindfulness-based smartphone app [27]. The magnitude of benefits was found to increase further following 30 days of use, with the rate of benefit greatest between baseline and day 10. The study did include a small sample size, an inactive control group, and inclusion of self-selected participants. A study by Ly et al showed that behavioral activation and mindfulness treatment helped adult participants who suffered from major depression [28]. Behavioral activation was found to be more effective for participants with a higher severity of depression, and treatment with mindfulness was more effective for patients who initially had a lower severity of depression. The study concluded that effective treatment of mild to moderate major depression through the use of smartphone app was feasible.

A study of college students with elevated stress levels used a mobile app daily; the majority of the intervention subjects indicated that the app helped with their stress and that they would use the app in the future [35]. The study concluded that delivering mindfulness meditation via an app is a more practical approach to reduce stress. This approach requires fewer resources, involves fewer time constraints, and allows students to participate remotely. A study by Bostock et al on healthy adult employees found long-term improvement in well-being, distress, job strain, and perceptions of workplace social support using mindfulness meditation delivered by a phone app [36].

Studies have shown the impact of EI and emotional regulation on individuals’ experiences of life and how changing the way a situation is perceived decreases its emotional impact [18]. The decreases in emotional experience and behavioral expression, which have no impact on memory, lower stress and improve relationships.

This study confirms the findings of previous studies that showed that mindfulness and EI training can be delivered via an app rather than requiring in-person instruction. To our knowledge, this is the first study to demonstrate that a text-based conversational app, Ajivar, can deliver personalized mindfulness and EI training with resulting improvements in anxiety, depression, and EI. In contrast to the previous work, EI and mindfulness training was delivered using AI and ML algorithms to personalize the user journey and experience. This study also demonstrated high user engagement with an app when learning techniques for mental wellness.

### COVID-19 Effect

Previous studies showed that college students experienced an uptick of anxiety and depression related to COVID-19, which were greater than the levels experienced previously during college [15,37,38]. In the Huckins [15] study, participants had almost a 50% increase in anxiety, as measured by the 2-item Generalized Anxiety Disorder (GAD-2) scale. The GAD-2 is a brief version of the GAD-7 questionnaire used in this study. When comparing GAD-7 scores in March with those at the start of the study, we showed an 11% increase in anxiety levels, though they did not reach statistical significance. With the use of Ajivar, these levels decreased to 55% (*P*=.001) below the GAD-7 scores at the start of the study among these individuals.

### Strengths and Limitations

Strengths of this study were the ability to use validated instruments to measure anxiety, depression, and EI in this sample. The study found positive results in a very limited amount of time. Limitations are evident in the study, including a small sample size. Some measurements were not available, as they were based on the usage of the app. For instance, students had to reach a certain level of usage to advance to the next area that may have provided additional data. There were no data available to control for influential variables external to Ajivar use. Lastly, the data are not generalizable.

### Future Studies

This was a small initial study, and increased participant numbers for future studies are anticipated. Future plans include a year-long study during the next academic year. The target population will be broader in scope and include students across the university, not restricted to specific classes.

### Conclusions

EI has proven key to long-term success. There is unlimited potential for using apps for mental health, including for issues related to the current pandemic. Also, similar studies should be replicated and completed to determine additional results of using mindfulness technology for mental health in a variety of populations, including college-age students. Technology apps can be used for mental health conditions along with other behavioral issues. Bakker et al found specific recommendations for mental health apps [39], including the aim to prevent emotional mental health problems. Also, to maximize engagement, it is recommended to use gamification, as was carried out in Ajivar, so that habit formation of self-care practices can take place [39]. Mental health apps have proven to provide positive results and success in a variety of populations. Additional testing is recommended as the pandemic continues. However, the results of this study demonstrate positive outcomes for mental and emotional health, along with a decrease in anxiety and depression levels. We are going to need scalable resources like Ajivar if we are going to meet the demands that we are likely to experience in the near future.

## Abbreviations

AI: artificial intelligence
ANCOVA: analysis of covariance
EI: emotional intelligence
EQ: emotional quotient
GAD-2: 2-item Generalized Anxiety Disorder
GAD-7: 7-item Generalized Anxiety Disorder
ML: machine learning
PHQ-9: 9-item Patient Health Questionnaire
